# Neglected Disease Research and Development: How Much Are We Really Spending?

**DOI:** 10.1371/journal.pmed.1000030

**Published:** 2009-02-03

**Authors:** Mary Moran, Javier Guzman, Anne-Laure Ropars, Alina McDonald, Nicole Jameson, Brenda Omune, Sam Ryan, Lindsey Wu

## Abstract

Mary Moran and colleagues survey global investment into research and development of new pharmaceutical products to prevent, manage, or cure diseases of the developing world.

The need for new pharmaceutical tools to prevent and treat neglected diseases is widely accepted [1]. The creation of a vaccine for HIV/AIDS, more effective diagnostics for tuberculosis (TB), and better treatments for leishmaniasis and sleeping sickness would greatly improve health in the developing world in line with the United Nations Millennium Development Goals. However, funders wishing to invest in this vitally important area currently face an information gap.

There is little consensus on what constitutes a neglected disease or what new products are required [2]. Health research funding figures have been published by the Council on Health Research for Development and the Global Forum for Health Research [3,4], but these do not disaggregate product-related research and development (R&D) or neglected disease investments.

Specific R&D investment data are available for some neglected diseases—including annual surveys of HIV/AIDS and TB funding since 2000 and 2005, respectively [5,6], and a one-off survey of malaria R&D funding published in 2005 [7]—but these cannot readily be compared since each survey uses different methodologies and covers different diseases, products, donors, and countries. For most neglected diseases, there is simply no information.

In order to address these information deficits, the Bill & Melinda Gates Foundation commissioned the George Institute for International Health to conduct five sequential annual surveys of global investment into R&D of new pharmaceutical products to prevent, manage, or cure diseases of the developing world. This article summarises key data from the first G-FINDER report (http://www.thegeorgeinstitute.org/prpppubs).

## Methodology

The chief principle of the G-FINDER survey was to provide consistent, comparable, comprehensive data on neglected disease R&D investments to those working in the field.

Survey design therefore focused on two elements. The first was disease, product, and research scope; the second was design of the survey tool that would be used to collect the data. Both were determined in consultation with an international Advisory Committee and Stakeholder Network (see Text S1 and S2).

The Advisory Committee included 17 international public health and product development experts who provided advice on inclusion and exclusion of diseases and products, and specialist knowledge of funders and developers in the neglected disease field. The Stakeholder Network included 25 major funders and product developers who advised on survey design, in particular on how to best achieve balance between the level of detail funders would find useful and the level they could feasibly provide. This network also provided input on issues to be managed in the design process, such as national and organisational variations in record-keeping and classification systems; multi-year disbursements; allocation of platform research and core funding between different diseases; overheads; double-counting of donor funding by intermediary or recipient bodies; and variations in financial years and currencies.

### Disease, product, and research scope.

In order to create maximum utility for funders, G-FINDER was designed to include all neglected diseases and products of significance to developing countries. This included not only Type III diseases, which are “overwhelmingly or exclusively incident in the developing countries”, but also developing-country presentations of Type II diseases, which are “incident in both rich and poor countries, but with a substantial proportion of the cases in the poor countries” and where R&D is “not in proportion to global need or addressed to the specific disease conditions of poor countries” [8]. Type I diseases, which occur commonly in both rich and poor countries, were excluded on the grounds that “the incentives for R&D exist in the rich country markets [and therefore]… products get developed” [8].

The first difficulty was to reach agreement on what constituted a neglected disease, since differing lists were used by the World Health Organization, the Commission on Macroeconomics and Health, the Global Network for Tropical Diseases, and a range of academic authors [8–12]. We therefore created a matrix of all Type II and III diseases that had been nominated by a major health organisation or publication as neglected diseases of the developing world, and all relevant products for each disease. A detailed list of R&D activities for each disease and product was also prepared based on previous categorisations developed by the United States National Institutes of Health (NIH) and the Wellcome Trust [13,14].

**Figure 1:**
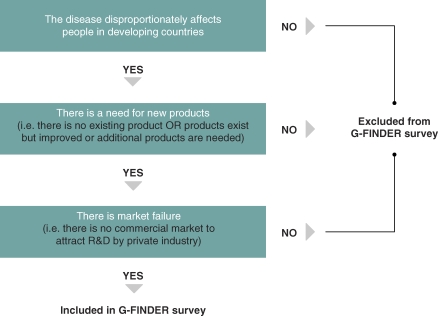
Three-Step Filter To Determine Scope of Neglected Diseases Covered by G-FINDER

These lists were then submitted to the Advisory Committee, who were asked to include or exclude diseases and products based on three filtering criteria (Figure 1). They were also asked to confirm or amend the detailed list of R&D activities for each product disease area. This was particularly important for diagnostics, vector-control products, and platform technologies, where development pathways for commercial and public health markets can differ.

This consultation process resulted in an agreed list of 30 neglected diseases and 127 neglected disease-product areas for which new product investment was needed (see Table 1), and a prescriptive list of which R&D activities were included and excluded.

**Table 1:**
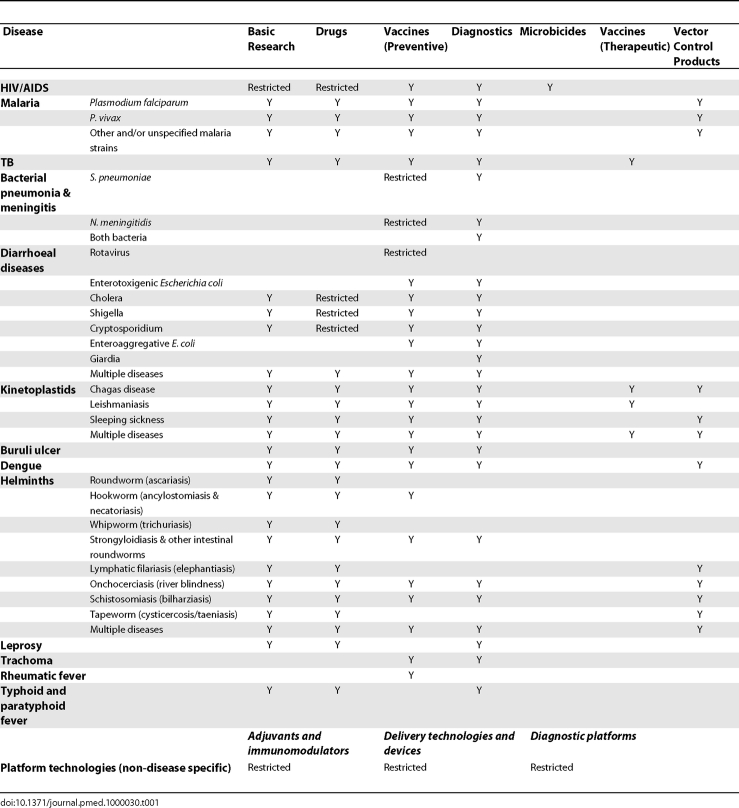
Diseases and Products Included in G-FINDER

Inclusion of investment data for some disease-product areas was “restricted”, that is, only developing-country-specific R&D investments could be included as defined in the detailed list of R&D activities. This was important to prevent neglected disease data being swamped by “white noise” from overlapping commercial R&D investments (e.g., in HIV and pneumonia); by investments in platform technologies with shared Western, as well as potential developing country, applications (e.g., adjuvants, diagnostic platforms); or by funding for activities not directly related to product development (e.g., advocacy, behavioural research).

The definition of what constituted a “developing-country-specific” investment for each restricted disease and product category was reached through an intensive consultation process over four months with the Advisory Committee. In some cases, views of additional disease experts were sought before consensus could be reached. This resulted in a tailored set of criteria for each restricted disease-product category, reflecting differences in disease and product profiles, research approaches, and products already available. The example of vaccines for bacterial pneumonia and meningitis is given below.

The Advisory Committee consensus was that vaccines for only two strains of bacterial pneumonia and meningitis (Streptococcus pneumoniae and Neisseria meningitidis) should be eligible for inclusion in G-FINDER. Within this, the Committee defined “developing-country-specific” products, for which investments could be included, as those that met the following criteria. For S. pneumoniae, the vaccine should be, at a minimum, designed for use in infants less than two years of age and provide coverage against S. pneumoniae serotypes 1, 5, and 14. For N. meningitidis, the vaccine should provide coverage against N. meningitidis serotype A, be a conjugate rather than a polysaccharide vaccine, be designed for use in infants less than two years of age, and be designed to cost less than a dollar per dose. For multivalent pneumonia vaccines covering Western and developing country strains, only developing-country-specific costs were eligible, defined as trials, registration, and phase IV/pharmacovigilance studies carried out in the target developing countries for the vaccine. (See Text S3 for reference document setting out criteria for eligible, ineligible, and restricted R&D investment for all disease, product, and research categories.)

Investment into HIV/AIDS for two categories, drugs and basic research, was also restricted to funding targeted to developing-country-specific presentations. For basic research, this was defined as research into mechanisms related to preventative vaccines and microbicides (e.g., immunological responses to potential antigens, mechanisms of mucosal transmission) but excluding general research that could also be applied to commercial products. Research into HIV drugs also included only developing-country-specific applications, such as label extensions to paediatric patient groups, fixed dose combinations, and slow release formulations. These restrictions were important to prevent developing-country-specific funding being swamped by the high level of public and private investment into HIV R&D targeted at Western needs.

### Survey tool and survey process.

As noted above, G-FINDER sought to provide data that were as consistent and comprehensive as possible across the range of neglected diseases surveyed, in order to enable analysis across diseases, funders, or regions and, as the survey is repeated, over time.

In order to achieve this, we followed two core principles. Firstly, only raw primary data reported by the funders, product development partnerships (PDPs), and product developers themselves were included in the survey. If these data were not available, they were not supplemented with secondary data or estimates. Secondly, in order to make the data as comparable and consistent as possible, all raw primary grant data were collected using the same online/offline reporting tool and inclusion/exclusion framework for all survey recipients. The only exception was the NIH, where a proportion of grants could not be collected in this way due to NIH data management system changes (see below on how exceptions were handled).

Survey participants were asked to enter every neglected disease grant they had disbursed or received in 2007 into a password-protected online database, including the grant amount, grant identification number, a brief description of the grant, and the name of the funder or recipient of the grant. They were also asked to confirm their organisation details such as role in funding (e.g., funder, fund manager, product developer), financial year, currency used, type of organisation (e.g., private sector firm, academic institution, PDP, multilateral organisation), and country where they were located. Each grant was entered using a three-step process where the survey recipient had to choose (1) a specific disease or sub-disease; (2) a product type (e.g., drugs, vaccines, microbicides); and (3) a research type within the product (e.g., discovery and preclinical, clinical development) according to pre-determined categories as described below. (See Text S4 for selected screen shots of the survey tool.) Where survey recipients could not provide data to this level of detail, they were asked to provide the finest level of granularity they could, ideally to at least disease and product level. The option of “Unspecified” was available in steps 2 and 3. If survey recipients were not able to allocate the grant to a single disease in step 1, three options were available:

- “Core funding of a multi-disease organisation” (e.g., funding to an organisation working in multiple diseases, where the expenditure per disease was not known to the funder);
- “Platform technologies”, further allocated as investment into diagnostic platforms; adjuvants, and immunomodulators; or delivery device platforms;
- “Other R&D” for any grants that still could not be allocated.

A separate reporting template was developed for multinational pharmaceutical companies, since these do not operate on a grants basis. Companies agreed to provide full data on their neglected disease investment. These data were defined as salary costs of full-time equivalent (FTE) staff employed on neglected disease programmes, including employment costs and benefits but excluding overheads; and all non-FTE direct project costs according to predefined lists developed by G-FINDER. These lists included typical R&D activities such as screening, medicinal chemistry, batch manufacture, and bioanalysis studies. Companies were asked to allocate their investments by disease, product and research type, according to the same guidelines used for online survey recipients.

We note that all participants agreed to provide actual 2007 grant disbursements and investments, as opposed to commitments made but not yet disbursed, or “soft” figures such as in-kind contributions, costs of capital, or funding estimates. Organisations submitted data based on their financial year, rather than the calendar year.

This system was varied in the case of the NIH. Due to restructuring of NIH information systems, only the Office of AIDS Research and the intramural arm of the National Institute of Allergy and Infectious Diseases were able to provide raw grant data. Taken together, these represented 68% of NIH 2007 expenditures on neglected disease R&D accounted for in the survey. On the advice of the NIH, the remaining 32% were obtained and filtered from public official databases.

Raw grant data for selected diseases were also shared with and between other survey groups (Families USA, Treatment Action Group, and the HIV Vaccines and Microbicides Resource Tracking Working Group) to avoid resurveying funders when possible. Any raw data received from other groups were reviewed, cleaned, and reclassified according to G-FINDER guidelines prior to entry into the database.

Data were entered over a 10-week period from July to September 2008, with a final total of 5,116 grants or investments recorded in the database.

### Data cleaning and analysis.

Survey closure was followed by a three-month period of intensive cleaning, cross-checking, and organising of the complex dataset collected.

Prior to commencing cross-checking, foreign currencies were converted to US dollars based on the 2007 average annual exchange rate as reported by the International Monetary Fund [15]. All non-NIH grants over US$0.5 million (i.e., any grant over 0.02% of total funding) were then verified through a three-step process. Each grant was first manually reviewed against our inclusion criteria to ensure only relevant R&D funding was captured. Over 3,000 grants were manually checked for correct allocation. Automated reconciliation reports were then used to cross-check self-reported funder data grant-by-grant against the funding reported as received by product developers and intermediary groups such as PDPs in order to identify inconsistencies. The final step was to resolve any discrepancies through direct contact with the funder and recipient to identify the correct figure. When reported amounts differed between funder and recipient due to practical factors such as differing financial years, the funder's amount was retained over that of the recipient. We note that these differences will be reconciled as the survey is repeated over the years. Industry figures were reviewed against industry portfolio information held by the George Institute and against FTE and direct costs provided by other companies. Costs that fell outside the expected range, for example, above average FTE costs for clinical staff, were queried and corrected with the company.

Analysis was conducted using automated reports constructed to show multiple data cuts from our cleaned and cross-checked database. These included calculating funding by disease, product, and research areas and possible combinations of these; and by funder, geographical region, recipient type, and so forth, with summary findings set out below. Industry figures were analysed at aggregate level, rather than by individual company. This included analysis of multinational pharmaceutical company aggregate contributions and small company aggregate contributions.

### Scope of recipients.

In its first year, G-FINDER sought to capture 2007 data from a wide range of funders and countries including:

- Public, private, and philanthropic funders in high- and middle-income countries that were part of the Organisation for Economic Cooperation and Development (OECD); all European Union Member States; and other high- and middle-income countries known to have an active research base (Singapore, Israel, and the Russian Federation)
- Public funders in selected innovative developing countries—IDCs (South Africa, Brazil).

In subsequent years, the survey will expand to include private sector funding in these two IDCs, as well as public funding in additional IDCs (India, China, Cuba) and other low- and middle-income countries.

Potential funders were identified through a number of avenues. The Health Policy Division has been working in the neglected disease field since 2004, during which time we have developed a large internal database of neglected disease funders, intermediaries, and product developers. This knowledge was built through scanning portfolios, media releases, industry reports, conference presentations, personal contacts, and in our role as Expert Advisors on a previous funding survey (the Malaria R&D Alliance survey of 2005). Previous neglected disease surveys have also collated lists of funders, intermediaries, and developers working in HIV, TB, and malaria; and we liaised closely with these survey groups, and with our Advisory Committee, to exchange information on known organisations in the field. Collation of this information resulted in a list of 551 organisations in the 43 target countries, who had previously recorded involvement in neglected disease product development. Of these, 134 were funders (the target group for the survey).

These 500-plus potential survey recipients were prioritised based on their R&D role (funder, developer, intermediary such as PDPs), level of funding, and area of disease and product activity.

Our top priority, as noted, was to target funders; however, it was known that some funders played a more pivotal role than others. For example, previous surveys had identified that 88% of malaria R&D funding [7]; 88% of HIV R&D funding [5]; and 83% of TB funding [6] were due to only 12 funders in each area, with these funders showing significant overlap across the three diseases. Based on published levels of funder investment in previous surveys, we were able to sub-divide the 134 funders into three groups. Maximum priority funders were defined as groups known to have previously invested over US$10 million dollars in neglected disease R&D funding annually; high priority funders were defined as groups known to have provided over US$5 million but less than US$10 million dollars; low priority were defined as groups providing less than US$5 million per year. The 43 participating countries (as opposed to individual funders) were also classified into three priority levels according to their gross expenditure on R&D (GERD) as a percentage of gross domestic product, as reported by the OECD [16]. The GERD ranking allowed us to identify countries that had not appeared in published neglected disease surveys, but whose high R&D expenditure might include domestic organisations with a previously unidentified component of neglected disease R&D.

All potential survey recipients were then reviewed for their activity in diseases outside HIV, TB, and malaria, to ensure the survey did not overlook groups who might only be active in other diseases and would therefore not have appeared in previous HIV, TB, and malaria funding surveys. Based on this review, on our internal database, and on advice from our Advisory Committee, we identified known or suspected major funders, PDPs, and product developers (public, academic, and private) for the remaining 27 G-FINDER diseases. If not already included, these were added to our high priority group.

This prioritisation process resulted in three survey strata, as follows. The maximum priority group included 25 organisations known to be major funders (over US$10 million per year) or major private sector developers investing internally in the 30 target neglected diseases. The high priority group of 85 organisations included known significant funders (US$5–US$10 million per year); potential research funders in high-GERD countries; and a range of academic research institutes, PDPs, government research institutes, multilaterals, multinational pharmaceutical firms, and small companies, who collectively provided good coverage of all disease areas. The remaining survey recipients, classified as low priority, were known very small funders or other known grant recipients, including many academic groups and public research institutions. We note that, although data from grant recipients were not included in the survey, they were collected to underpin the cross-checking process and track funding flows through the system. In addition, they provided extremely useful insights into how and where neglected disease research is conducted.

The G-FINDER process focused on the 110 organisations included in the maximum and high priority groups, who likely represented the majority of global neglected disease R&D funding. Substantial pro-active effort was put into securing high survey completion and data accuracy rates from these groups. The remaining groups were passively surveyed, but not pro-actively pursued for information.

We received complete financial information from 150 organisations. In the maximum priority group, 23 of 25 recipients (92%) provided funding information for 2007. Two maximum priority organisations, Wyeth-Ayerst Lederle and Merck (both multinational pharmaceutical companies), did not provide data; and one, sanofi-aventis, provided incomplete data (drug but not vaccine data). In the high priority group, 77 organisations (91%) provided full funding information for 2007.

## Survey Findings

### Overall funding.

Just over US$2.5 billion was invested into R&D of new neglected disease products in 2007.

Funding was highly concentrated, with HIV/AIDS, TB, and malaria receiving nearly 80% of the total (see Table 2). Other equally high-burden diseases as measured by DALYs (disability-adjusted life years), such as pneumonia and the diarrhoeal illnesses, collectively received less than 6% of total funding.

**Table 2:**
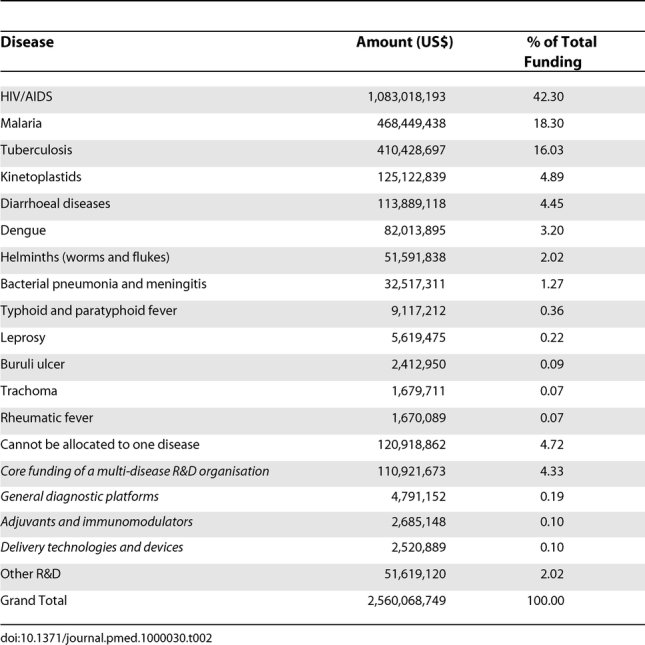
Total R&D Funding by Disease

Overall, product R&D investment was heavily focused on drugs and vaccines. Investment in new diagnostics was patchy, while platform technologies applicable to many diseases, for instance vaccine adjuvants, diagnostic platforms, and delivery technologies, received less than 0.4% of total R&D investment.

### Funding for “the big three”: HIV/AIDS, malaria, and TB.

**Figure 2:**
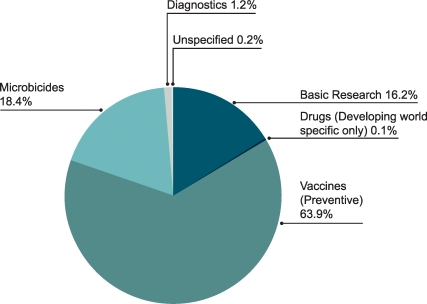
HIV/AIDS R&D Funding in 2007

Total R&D funding for HIV/AIDS was US$1.08 billion, with US$692 million (63.9%) directed to vaccines, US$200 million (18.4%) to microbicides, and US$176 million (16.2%) to basic research. Less than US$1 million (0.1%) was invested in development of HIV drugs targeted at developing world needs, such as paediatric formulations and fixed-dose antiretroviral combinations (Figure 2).

**Figure 3:**
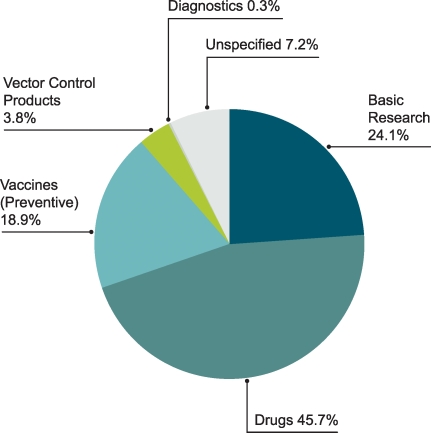
Malaria R&D Funding in 2007

Total funding for malaria was US$468.5 million, with drug development receiving nearly half this amount (US$214 million or 45.7%). Basic research was the next highest funded category at US$113 million (24.1%), while vaccines received US$88.4 million (18.9%). Vector control products, such as insecticides and biological control measures, received US$17.7 million. Malaria diagnostics received very limited funding of US$1.6 million (0.3% of the total) (Figure 3).

**Figure 4:**
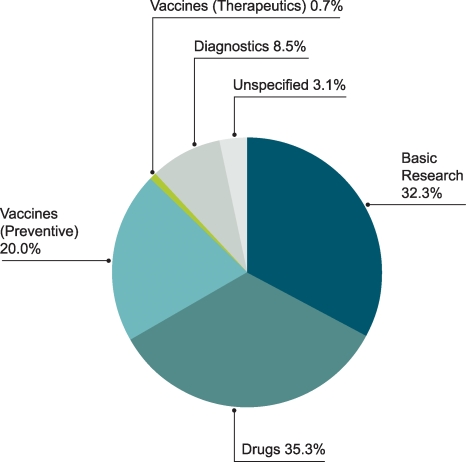
TB R&D Funding in 2007

TB funding for 2007 totalled US$410.4 million. As with malaria investments, the bulk of this went to R&D of TB drugs (US$145.1 million or 35.3%), closely followed by basic research at US$132.4 million (32.3%). TB vaccines were funded at similar levels to malaria vaccines in both actual and relative terms, receiving US$82.3 million (20%) of total TB funding. However, TB diagnostics fared dramatically better, receiving 8.5% of global TB investment (US$35 million) (Figure 4).

## Neglected Disease Funders: Who Are They?

### Funding by sector.

**Figure 5:**
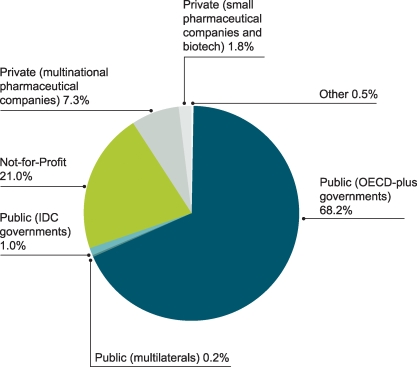
Total R&D Funding by Funder Type in 2007

Neglected disease funding remains primarily the realm of public and philanthropic donors, who collectively invested US$2.3 billion or 90% of the total funding in 2007 (Figure 5).

Public donors, including government and multilateral groups, provided US$1.78 billion (69%) while philanthropic and not-for-profit funders invested US$538 million (21%). Although only two IDCs were included in year one of the survey, they represented 1% of global spending. IDC figures are expected to be substantially larger in subsequent G-FINDER surveys due to the inclusion of India, China, and Cuba.

The private pharmaceutical industry provided around 9% of the global total, with an aggregate investment of US$231.8 million. This collectively made the pharmaceutical industry the third largest global investor in neglected disease R&D behind the NIH and the Bill & Melinda Gates Foundation. Small companies and biotechs represented around 20% of reported industry investment (US$46.2 million), with multinational firms contributing US$185.7 million (80%).

### Funding by government.

The US Government represented nearly three quarters of global public spending with an investment of US$1.25 billion (70%) through its various institutes and departments (Table 3). European Governments and the European Commission collectively provided US$384.9 million (22%), with the UK, the Netherlands, Republic of Ireland, and Sweden dominating the field. The increasing role played by some non-G7 economies was notable, with Brazil ranking as the 6th largest government funder and Russia as the 10th.

**Table 3:**
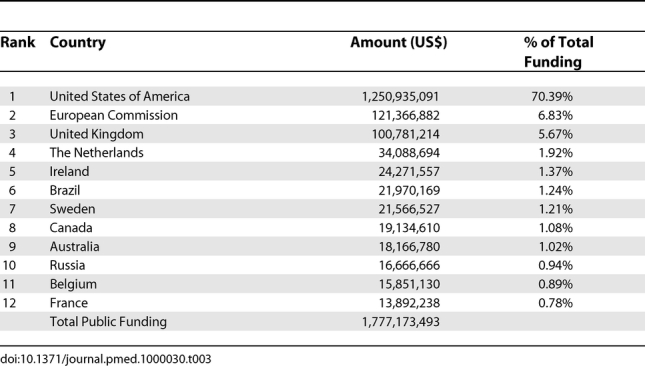
Top 12 Government/Public Funders

### Funding by organisation.

It was also illuminating to examine funding from the perspective of individual organisations. This showed that global investment into creating new neglected disease products is heavily reliant on a mere handful of donors. Twelve organisations provided around 80% of global funding, with the NIH and the Bill & Melinda Gates Foundation collectively investing US$1.51 billion or 59.5% of the total (Table 4).

**Table 4:**
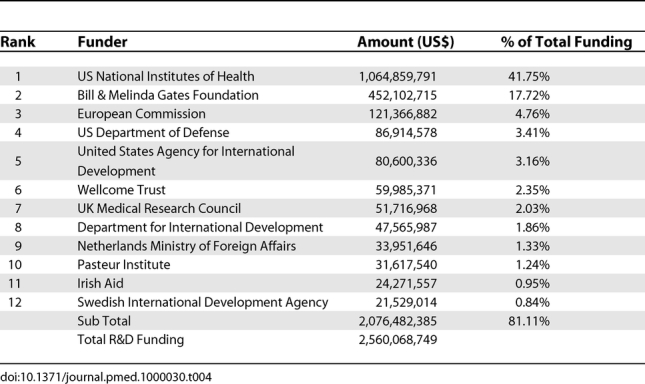
Top 12 Funders by Organisation

For confidentiality reasons, industry investments were aggregated. However, we note that if this had not been the case, some pharmaceutical companies would have appeared in the list of top 12 funders based on the size of their internal investments.

## Funding Recipients: Who Are They?

Neglected disease R&D is conducted by a wide variety of organisations, including academic groups, public institutions, and private companies. Some rely entirely on external grants for their neglected disease R&D programmes, while others fund their programmes from their own budgets, and yet others operate on a mix of both.

Funders also appeared to increasingly rely on PDPs to manage and allocate funding (Table 5). Twenty-three percent of donor funding was routed via PDPs, who reallocated it to industry and academic partner programmes, rather than being granted directly by donors to recipient organisations.

**Table 5:**
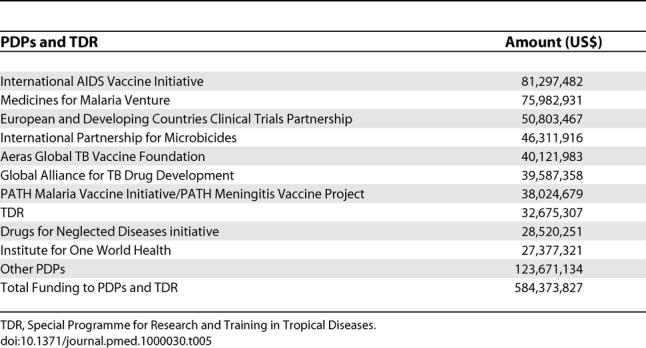
Funds Received by PDPs and TDR

## Discussion

### Limitations to the methodology.

G-FINDER is, we believe, the most rigorous neglected disease R&D survey conducted to date in terms of its scope, methodology, and breadth of participation. Strenuous efforts were made to reach agreement on definitions of diseases, products, and research activities for inclusion. Equal effort was put into designing a data collection system that provided an identical reporting framework for each organisation, despite differences in currency, financial year, research categorisation, and reporting systems. By collecting primary raw grant and investment data in the same way from all organisations, we believe the G-FINDER survey allows for a more accurate comparison of funding across the range of neglected diseases than has been possible before.

However, it is also important to note the limitations of the survey. The greatest limitation is survey non-completion, particularly by maximum priority groups who are by definition major funders or play a significant R&D role in a specific disease-product area. We believe that data from *public* maximum priority funders are close to 100% complete, since all identified public groups provided full data. However, private sector figures are almost certainly under-reported due to the non-participation of two maximum priority multinational pharmaceutical companies and provision of incomplete data by another. We note that at least one of these companies has a late-stage pneumonia vaccine programme for commercial markets; however, this may have included some investment in developing country studies that will have been missed. One of the three firms also had a late-stage HIV vaccine in development, which will have led to significant under-reporting of AIDS vaccine investments.

A second area where missing private sector data are likely to distort results is that of diagnostics, where smaller companies play an active role. A total of 150 small firms were identified as having neglected disease R&D activity, many of them working in the diagnostic field, particularly HIV and TB diagnostics; however, only 28 of these provided data for the survey. The plethora of small firms in the diagnostic field is likely to reflect the substantially lower development cost and time of bringing new diagnostics to market, compared to developing new drugs and vaccines. Low participation rates by these small firms mean diagnostic investments are likely to be under-reported, particularly in HIV and TB. As a result, the diagnostic funding reported in G-FINDER for these two diseases needs to be treated with caution.

It is also important to note other factors that are likely to have led to under-estimation of the value of industry investments. Companies who participated in the survey agreed to exclude costs that were difficult to quantify in order to avoid degrading the quality of data—for instance, costs of capital, infrastructure costs, and the value of in-kind contributions. Although difficult to quantify, these inputs nevertheless have a substantial value and represent a substantial unrecognised cost to companies.

Differing level of responsiveness between organisations and countries may also skew findings. For instance, it is possible that the Australian location of the G-FINDER group led to high levels of responsiveness from Australian funders, or that funders in non-English-speaking settings were less enthusiastic in their levels of response. This is not known to be the case, but is simply noted as a possible factor.

Finally, G-FINDER can only report the data that were given to us. Although strenuous efforts were made to check the classification, accuracy, and completeness of grants, in a survey this size it is likely that some data will still have been incorrectly entered or that donors may have accidentally omitted some grants. The requirement to use public official databases for some NIH data, as opposed to raw grant data, also means these figures may contain inaccuracies or omissions that we were unable to detect. We believe, however, that the checks and balances built into the G-FINDER process mean that such mistakes, if present, will have a minor impact.

### Observations.

The G-FINDER survey is intended to report accurate, comparable R&D investment figures across the spectrum of neglected diseases. We are not seeking to analyse whether investment is best spent, or to make recommendations on funding allocation; nor are we attempting the task of ascertaining how much R&D funding is “enough”. Although we acknowledge the importance of these questions, it would require a research effort of equal or greater scope to the task undertaken here. The G-FINDER report is simply the first step in the process, providing information to allow discussion and decision-making to begin.

We therefore restrict ourselves to a few observations. The first is that research funding is highly concentrated and has little correlation with burden of disease, as measured by DALYs.

For instance, HIV, TB, and malaria accounted for 125 million DALYs in low- and middle-income countries in 2004 [17] and received nearly 80% of total funding; while pneumonia and the diarrhoeal illnesses accounted for 165 million DALYs in these countries in the same year [17] but received less than 6% of total funding. Likewise, helminth infections received less than half the funding of kinetoplastid diseases although their disease burden was three times higher (12 million DALYs in 2004 compared to 4 million DALYs for the kinetoplastid diseases) [17]; while dengue had a disease burden 20 times lower than helminth infections at 600,000 DALYs in 2004 [17] but received nearly twice as much funding (see Table 2).

This observation should not be construed as a statement or belief that funding levels can or should be based only on disease DALYs. DALYs are the most widely accepted quantitative indicator of burden of disease, but remain an imperfect measure. For some diseases, such as Buruli ulcer, reliable DALYs are virtually nonexistent; correct estimates can be difficult to obtain in regions with weak data; and DALYs do not reflect the impact of co-morbidities, such as HIV and TB. The DALY comparison is noted here chiefly to give a sense of the differences in scale of funding for different diseases.

It is important to note, however, that funding levels reflect many factors beyond DALYs. The current state of science for a given disease plays a role, since many would-be investors in product development look at feasibility as well as need. Conversely, other investors may specifically choose to invest in areas where science is lacking, believing their best contribution will be funding a breakthrough discovery to unlock the path to new products.

Funders may also look at the state of existing therapies, preferring to focus on areas where no safe effective treatments exist (for example, some kinetoplastid diseases) to areas where treatments exist even though they may be limited or sub-optimal (for example, some helminth infections). Funders may also want to focus on diseases with a higher relative or absolute mortality, such as dengue or meningitis, rather than diseases with lower relative mortality but a higher DALY burden.

The type of R&D needed in a given disease area is also a crucial factor in interpreting the data, since R&D costs vary dramatically depending on the kind of product being developed and how far down the development pathway that product is. Vaccines are far more expensive to develop than drugs, and drugs than diagnostics. Clinical development is also far more expensive than pre-clinical or discovery research. For instance, out-of-pocket development cost of a diagnostic test can be in the low tens of millions; while clinical development cost of a novel fixed-dose combination anti-malarial is estimated at US$30 million, and of a malaria vaccine at just over US$100 million [18]. Thus, far more investment would be needed to develop a meningitis vaccine than a TB diagnostic, irrespective of the lower burden of disease for meningitis or a more advanced state of meningitis vaccine technology.

Despite this, the concentration of funding on AIDS, TB, and malaria nevertheless suggests that investment decisions are not only influenced by scientific or epidemiological considerations, but may also be influenced by factors such as the presence of PDPs or civil society groups with active advocacy, fundraising, and investment activities; by funder perceptions or preferences; or by the presence of policy frameworks and funding mechanisms that prioritise specific diseases.

The predominance of research into new products for HIV/AIDS, malaria, and TB is understandable—and the generosity of funding in these areas is a credit to donors—however, other high-burden, high-mortality diseases remain badly under-funded: pneumonia and the diarrhoeal illnesses stand out in this regard. The tendency to focus on drugs and vaccines over diagnostics, platform technologies, and developing-country-specific products is also of concern, particularly as these latter categories can represent a highly effective use of resources since any advance will be applicable to many neglected diseases and products.

A second observation relates to the concentration of funding. The participation of so many organisations and countries in development of new neglected disease products is a remarkable and welcome change from past decades of inertia and neglect. It is clear, however, that these efforts are not evenly distributed, with several major OECD governments missing in action from the top 10, top 20, or even the top 50 funders of R&D for neglected diseases. It is also remarkable that investment by some private firms is now rivalling or exceeding spending by many public organisations, and indeed many G7 and OECD countries. While we commend these companies and philanthropists, their efforts are meant to support, not replace, those of wealthy governments around the world.

A broadening of funding efforts so that all who are able to contribute do so, and all diseases receive the attention they deserve, would lead to a dramatic positive impact on the health of developing country patients afflicted with these diseases. We very much hope that the information presented in the G-FINDER report will contribute to this process.

## Supporting Information

Text S1 G-FINDER Advisory Committee (40 KB DOC).

Text S2 G-FINDER Stakeholder Network (41 KB DOC).

Text S3 G-FINDER R&D Reference Document (288 KB PDF).

Text S4 G-FINDER Online Survey: A Sample of Screenshots (617 KB DOC).

## Abbreviations

DALY: disability-adjusted life year
FTE: full-time equivalent
GERD: gross expenditure on R&D
IDC: innovative developing country
OECD: Organisation for Economic Co-operation and Development
NIH: National Institutes of Health
PDP: product development partnership
R&D: research and development
TB: tuberculosis
