# Supplementary material for: Neglected Disease Research and Development: How Much Are We Really Spending?
Source: PLoS Med. 2009 Feb 3;6(2):e1000030. doi: 10.1371/journal.pmed.1000030 (PMC2634791; doi:10.1371/journal.pmed.1000030)
Supplement: Supplementary file 1 [file pmed.1000030.sd003.pdf]

**RESEARCH AND DEVELOPMENT REFERENCE DOCUMENT**


Basic research

Drugs

Preventative vaccines

Diagnostics

Microbicides

Therapeutic Vaccines

Vector Control Products

Cannot be allocated to one disease

- Core funding of a multi-disease R&D organisation
- Adjuvants and immunomodulators
- Delivery technologies and devices
- General diagnostic platforms
- Other R&D

Out of scope (excluded from the survey)

- General exclusions
- Non-product R&D
- Selected disease and product restrictions

# I BASIC RESEARCH

*Studies that increase scientific knowledge and understanding about the disease, disease processes, pathogen or vector, but which is not yet directed towards a specific product*

## 1 NATURAL HISTORY AND EPIDEMIOLOGY

1.1 Basic mechanisms of disease transmission

1.2 Disease prevalence in relation to human genotype, strain variation, and inoculation rates

1.3 Genetic diversity and phylogeny

1.4 Epidemiological research on the roles of human behaviour and effects of specific host genotypes on disease transmission

1.5 Epidemiological research on host genetic factors influencing the prevalence of disease (e.g., sickle cell, HLA type, Rh factor) or the impact of disease in select host genotypes

1.6 Epidemiological research on the distribution of pathogen, vectors and the prevalence of morbidity and mortality due to the disease that is NOT related to specific product development

1.7 Epidemiological research on antigenic variability; population studies of human immunity to the disease

1.8 Epidemiology of drug resistance or evolutionary studies on resistance development for established, existing drugs

1.9 Epidemiological research related to vector behaviour & ecology, and vector control

## 2 IMMUNOLOGY OF DISEASE

2.1 Defining signalling pathways of immune function (mechanisms of systemic and/or mucosal immunity)

2.2 Interaction and impact of the signalling pathways with the pathogen

2.3 Development of assays or tools potentially useful for drug, vaccine, or microbicide research & development

2.4 Identification of immune correlates of protection, including *in vivo* and *in vitro* studies on the protective immune response (cellular, humoral, and/or mucosal)

2.5 Investigating the immune response to particular antigens; studies of specific antigens or immunogens proposed as vaccine candidates

2.6 Development of animal models to determine immune correlates of protection and to assist in vaccine design and testing

2.7 Genetics of the immune response to the disease & effects of antigen polymorphism or genetic diversity on specific vaccine candidates (as recognised from field studies)

## 3 BIOLOGY OF DISEASE

3.1 Structure and morphology of different developmental stages

3.2 Host–parasite interactions and biology of pathogen interaction with vector host

3.3 Biology of invasion of host cells (entry mechanisms)

3.4 Localisation of pathogen proteins or antigens

3.5 Development of culture and purification tools to assist in study of the pathogen

3.6 Descriptions of pathogenic species and characterisation of strains or subtypes in animal models (course of infection, susceptibility of different hosts)

3.7 *In vitro* studies of interactions between the pathogen and other infectious agents (e.g., Epstein-Barr virus)

## 4 BIOCHEMISTRY OF THE PATHOGEN

4.1 Metabolism and nutrition

4.2 Protein sequencing, enzymology, and protein and enzyme characterisation (including antigen analysis)

4.3 Signal transduction; translation, processing and export of proteins

4.4 Glycosylation, GPI anchors, transporters, ion channels, mitochondrial metabolism, and electrophysiology studies

4.5 Influence of pathogen on host-cell biochemistry

4.6 Characterisation of antigen/protein diversity of pathogenic strains and subtypes

4.7 Characterisation of proteins and molecular basis for host-cell invasion

4.8 Analysis & characterisation of drug-resistant strains and studies probing drug resistance mechanism/s or pathways

4.9 Non-specific research on pathogen or host targets to identify potential drug, vaccine, or diagnostic targets (i.e., target identification)

## 5 GENETICS OF THE PATHOGEN

5.1 Studies on chromosomes; genomic maps; genetic crosses

5.2 Cloning and sequencing of genes; cDNAs for functional proteins (including drug targets and vaccine candidates)

5.3 Expression of proteins from cloned genes; RNA analyses

5.4 Control and timing of gene expression; post-transcriptional processing

5.5 Analysis and characterisation of genes involved in drug resistance

5.6 Genetics of antigenic variability

5.7 Techniques for the genetic transformation of the pathogen

5.8 Tests for genotyping the pathogen for laboratory use

## 6 BIOINFORMATICS AND PROTEOMICS

6.1 Microarray analysis

6.2 Genome annotation – gene predictions

6.3 Comparative genomics, sequence alignment, genome assembly

6.4 Variation, single nucleotide polymorphisms (SNPs)

6.5 Database applications, data mining tools

6.6 Structural and functional genomics

6.7 Structural and functional proteomics

6.8 Proteome analysis, protein structure alignment

## 7 PATHOPHYSIOLOGY AND DISEASE SYMPTOMS

7.1 Clinical diagnosis and clinical observations of the disease presentation and pathophysiology in humans and in animals

7.2 The role of nutritional status in determining disease severity and treatment effectiveness

7.3 Histopathology of the disease in humans and in animals

7.4 The mechanisms of pathology of the disease; including, the role of the host immune system, and expression of adhesion molecules

7.5 Development of improved animal models to study disease pathophysiology, to evaluate the biological properties of drugs & microbicides

7.6 Identification of biomarkers for diagnostics or therapeutic monitoring

7.7 Studies of the mechanisms by which particular susceptible/resistant mammalian host genotypes exert their effect

7.8 Research on effects of host co-morbidities and secondary effects of pathogen invasion (e.g., research on anaemia /neurological effects of malaria)

7.9 Interactions between the disease and other relevant concurrent infections, including determining timing and establishment of infection

### 8 VECTOR BIOLOGY, BIOCHEMISTRY, AND GENETICS

8.1 Characterisation of vector behaviour & ecology

8.2 Studies of vector susceptibility to infection; studies of parasites and pathogens of vectors (including potential biological control agents)

8.3 Identification of genes responsible for disruption of parasite/virus growth, genetic transformation of vectors, & insect transposable elements

8.4 Target identification of vector sites that may become the subject of *in vitro* screening or molecular design

8.5 Development of tests for vector identification, taxonomy and systematics, and for the identification of infected vectors

8.6 Studies evaluating resistance development, including the genetics and transmission of pesticide resistance

## II DRUGS

*Research activities and processes necessary to develop and improve new compounds specifically designed to cure or treat neglected diseases; including drug discovery or design, preclinical and clinical development and other activities essential for successful drug development and uptake*

### 9 DISCOVERY AND PRECLINICAL

*Research activities targeted at discovering and optimising investigational compounds and including the processes needed to allow new chemical entities to proceed to human trials; including:*

9.1 Target validation, characterisation, & selection

9.2 High throughput screening, lead optimisation

9.3 Development of analytical tests for assaying drugs

9.4 Research on drugs from natural products; identification and characterisation of active ingredient

9.5 Research on the effects of drug treatment on immune status

9.6 Measurement of the activity of potential drugs *in vitro* and in animal models; including safety and efficacy studies necessary to satisfy Investigational New Drug (IND) requirements

9.7 Studies evaluating the activity of new drugs on drug-resistant strains, their effect on genes involved in drug resistance, or their effect on resistance pathways

9.8 Development of tests for drug susceptibility of the pathogen for research purposes

9.9 Drug pharmacokinetic, toxicity and metabolism studies *in vitro* and in animal models, including bioavailability, adsorption, metabolism, and excretion (ADME) studies

9.10 Chemistry and synthesis of drugs, including process and scale-up manufacture, production of GLP- and GMP-batch for toxicology studies; and other Chemistry and Manufacture Control (CMC) activities required to allow new chemical entities to proceed to human trials

9.11 Preparation of Investigational New Drug (IND) application for regulatory submission

9.12 Optimisation and manufacturing of new formulations to support label-extension[1] for new patient sub-populations (e.g., infants, pregnant women)

## 10 CLINICAL DEVELOPMENT

*Research activities and processes associated with clinical testing of investigational new drugs so as to demonstrate safety and efficacy in human subjects (as needed for regulatory approval), together with other costs required to support such clinical trials; including:*

10.1 Phase I pharmacokinetic, toxicity and metabolism studies in healthy human subjects

10.2 Phase II testing of drugs and drug combinations in human patients to establish safety and efficacy including dose-finding, and proof of principle studies

10.3 Phase III clinical trials for registration purposes

10.4 Regulatory standard clinical trials that support a formal registration for label-extension of an existing drug to a new disease or patient group (e.g. paediatric patients, pregnant women or HIV-positive patients)

10.5 Regulatory standard clinical trials that support formal registration for label-extension of an existing drug to a new use, such as intermittent preventative therapy and pre-exposure prophylaxis

10.6 Assessment & validation of trial sites to carry out a specific product trial

10.7 Infrastructure and site development costs directly associated with the conduct of clinical trials for drug development *in endemic countries* (e.g. refurbishment of hospital wing, vehicle purchase, generators, training and community relationship building)

10.8 Further pharmaceutical development to generate the final clinical formulation, dosage form and other Chemistry and Manufacture Control (CMC) activities required for regulatory submission

10.9 Compiling of all non-clinical and clinical data for submission of a New Drug Application (NDA) to regulatory authorities

10.10 Behavioural research *during clinical trials* relating to risk assessment, factors affecting adherence to protocol, and product acceptability

10.11 Protocol development, investigator meetings, GCP-monitoring, quality control, data management, analysis and reporting, establishing a Data Safety Monitoring Board (DSMB), and trial audits

[1] Label-extensions refer to changes to drugs or their labels after they have been approved. This includes changes in manufacturing, recommended patient population and/or formulation. To change a label, market a new dosage or strength of a drug, or change the way a drug is manufactured, the Company must submit a supplemental new drug application (sNDA) to regulatory authorities to obtain marketing approval.

### 11 PHASE IV/PHARMACOVIGILANCE

*Studies relating to the detection, monitoring, evaluation, and prevention of adverse events associated with newly approved drugs so as to bridge the gap between highly controlled clinical trials intended for regulatory approval and the largely uncontrolled use of new drugs by patients. Also includes studies conducted after regulatory approval that assess drug effectiveness in the wider population or which are necessary to support product use in developing countries.*

11.1 Pharmacovigilance and post-registration studies of newly registered drugs to assess adverse reactions, toxicology and safety

11.2 Effectiveness studies and head-to-head comparator studies of newly registered drugs (with other therapies or interventions)

11.3 Cost-effectiveness studies of newly registered drugs

11.4 Treatment interactions and population level studies (of newly registered products e.g., pharmaco-epidemiological and resistance studies)

11.5 Behavioural research *post-registration* of new drugs relating to risk assessment, factors affecting adherence to protocol, provider compliance, and product acceptability

11.6 Case history reports and assessment of long-term prophylaxis using newly registered drugs in communities in endemic areas

### 12 BASELINE EPIDEMIOLOGY

*Studies evaluating potential trial site populations to confirm disease incidence, prevalence or exposure risk, and which serve as the foundation for determining the optimal collection, analysis, interpretation and presentation of clinical trial data, including:*

12.1 Epidemiological studies directly linked to the conduct or support of clinical trials of products in development, in order to assess or validate the epidemiology of disease, disease incidence, or health of target populations at trial sites

12.2 Preliminary studies of morbidity & mortality at potential clinical trial sites, where these studies are directly linked to planned product trials

## III PREVENTIVE VACCINES

*Research activities and processes necessary to develop and improve investigational vaccines specifically intended to **prevent** infection; including vaccine design, preclinical and clinical development and other activities essential for successful vaccine development and uptake*

### 13 DISCOVERY AND PRECLINICAL

*Research activities targeted at discovering and optimising investigational vaccines and including the processes necessary to allow a candidate vaccine to proceed to human trials; including:*

13.1 Studies supporting novel vaccine design, including target validation & candidate optimisation

13.2 Evaluation of vaccine technologies (e.g. adjuvants, delivery systems) to improve the immunogenicity of an identified candidate

13.3 Pre-clinical safety and immunogenicity studies with candidate vaccines, including use or development of functional assays

13.4 Pre-clinical animal studies, challenge models and addressing the correlation between *in vitro* models, animal models and field results

13.5 Studies on the genetics of the immune response to selected antigens as vaccine candidates, optimisation of animal models and correlates to clinical results

13.6 Manufacturing scale-up and consistency of manufacture, including production of GLP and GMP-batches for regulatory toxicology studies and other Chemistry and Manufacture Control (CMC) activities required to allow a candidate vaccine to proceed to human trials

13.7 Research on safety and regulatory considerations (e.g. validation of preclinical assays to permit registration)

13.8 Preparation of an Investigational New Drug (IND) application for regulatory submission

13.9 Optimisation of vaccine candidates for global use (cheaper, more stable, ease of administration, addition of developing country-specific strains)

## 14 CLINICAL DEVELOPMENT

*Activities and processes associated with clinical testing of investigational vaccines so as to demonstrate safety, immunogenicity and efficacy in human subjects (as needed for regulatory approval), together with other costs required to support such clinical trials, including:*

14.1 Phase Ia studies assessing safety, dosing, and immunogenicity in human volunteers; Phase Ib studies assessing safety, dosing, and immunogenicity in clinically exposed or high-risk populations

14.2 Phase IIa challenge studies; Phase II safety and preliminary efficacy studies in exposed populations or those at high-risk of infection

14.3 Phase III expanded efficacy, effectiveness and safety studies required for registration purposes, including implementation, retention and follow-up of volunteers

14.4 Assessment & validation of trial sites directly linked to preparation for product trials

14.5 Infrastructure and site development costs associated with the conduct of clinical trials for vaccine development *in endemic countries* (e.g. refurbishment of hospital wing, vehicle purchase, generators, training and community relationship building)

14.6 Further biological/product development to generate the optimal clinical formulation and dosage form, and other Chemistry and Manufacture Control (CMC) activities required for regulatory submission

14.7 Compiling all non-clinical and clinical data to obtain a Biologics License from regulatory authorities

14.8 Protocol development, investigator meetings, GCP-monitoring, quality control, data management, analysis and reporting, establishing a Data Safety Monitoring Board (DSMB) and trial audits

## 15 PHASE IV/PHARMACOVIGILANCE

*Studies relating to the detection, monitoring, evaluation, and prevention of adverse events associated with newly approved vaccines so as to bridge the gap between highly controlled clinical trials intended for regulatory approval and the largely uncontrolled delivery of new vaccines. Also includes studies conducted after regulatory approval that assess vaccine effectiveness in the wider population or which are necessary to support product use in developing countries.*

15.1 Pharmacovigilance and post-registration studies of newly registered preventative vaccines to assess adverse reactions, toxicology and safety

15.2 Effectiveness studies and head-to-head comparator studies of newly registered preventative vaccines (with other therapies or interventions)

15.3 Cost-effectiveness studies of newly registered preventative vaccines

15.4 Treatment interactions and population level studies (of newly registered preventative vaccines e.g., pharmaco-epidemiological and resistance studies)

15.5 Behavioural research *post-registration* of new preventative vaccines relating to risk assessment, factors affecting adherence to protocol, provider compliance, and product acceptability

15.6 Case history reports and assessment of long-term prophylaxis using newly registered preventative vaccines in communities in endemic areas

### 16 BASELINE EPIDEMIOLOGY

*Studies evaluating potential trial site populations to confirm disease incidence, prevalence or exposure risk, and which serve as the foundation for determining the optimal collection, analysis, interpretation and presentation of clinical trial data, including:*

16.1 Epidemiological studies directly linked to the conduct or support of clinical trials of preventative vaccines in development, in order to assess or validate the epidemiology of disease, disease incidence, or health of target populations at trial sites

16.2 Preliminary studies of morbidity & mortality at potential clinical trial sites, where these studies are directly linked to planned preventative vaccines trials

## IV DIAGNOSTICS

*Research activities and processes necessary to develop, optimise, and validate diagnostic tests for use in resource-limited settings (cheaper, faster, more reliable, ease of use in the field); including discovery and design, preclinical and clinical evaluation, and other activities essential for successful deployment for public health use.*

### 17 DISCOVERY AND PRECLINICAL

*Research activities targeted at discovering and optimising low-cost, stable, easy-to-use diagnostics for neglected diseases and including the processes necessary to allow a potential product to proceed to clinical evaluation including:*

17.1 Validation, characterisation, and selection of targets suitable for diagnostic use

17.2 Validation of new diagnostic markers or biomarkers

17.3 Development and testing of low-cost, stable, easy-to-use diagnostic tests (e.g. simpler microscopy, improved sample collection/preparation, cheaper ELISAs), including manufacturing design

17.4 New or improved diagnostics for disease staging and therapy decisions

17.5 New or improved diagnostic tools to identify resistant pathogens

17.6 New or improved diagnostics to identify specific target populations

17.7 Tailoring diagnostic tools for developing country use, including improved point-of-care tests (rapid test), local laboratory test, reference laboratory tests and central laboratory tests

17.8 Creation of reference material banks

### 18 CLINICAL EVALUATION

*Activities and processes associated with clinical evaluation of investigational diagnostic tools so as to demonstrate sensitivity and specificity in human subjects, together with other costs required to support such clinical trials; including:*

18.1 Clinical efficacy trials

18.2 Small-scale testing in humans to establish sensitivity and specificity and utility

18.3 Technical evaluation of tests and studies evaluating product performance

18.4 Establishment of product specifications, kit development and quality assurance

18.5 Submission of relevant data to regulatory authorities for approval

18.6 Assessment & validation of trial sites to carry out product trials

18.7 Infrastructure and site development costs directly associated with the conduct of clinical trials for diagnostic development *in endemic countries* (e.g., refurbishment of hospital wing, vehicle purchase, generators, training and community relationship building)

### 19 Operational research

*Operational procedures and implementation activities associated with novel diagnostic tools, which are necessary to support WHO recommendation for global public health use including:*

19.1 Larger-scale demonstration studies (assessing specificity, sensitivity and utility of the diagnostic test in endemic countries)

19.2 Cost-effectiveness studies

19.3 Identification of pitfalls of the technology and studies of safety measures needed to support the technology

19.4 Studies to determine at what level of the health care system the technology is applicable (e.g. reference labs, regional labs)

19.5 Identification of training needs

19.6 Collecting evidence for expanding the use of a diagnostic tool in different countries

19.7 Development of equipment and customer support documents

19.8 Head-to-head comparator studies (with current gold standard) and in the context of existing diagnostic algorithms

19.9 Behavioural research relating to risk assessment, factors affecting diagnostics use, and user acceptability (patient and provider)

19.10 Epidemiological studies to assess or validate the epidemiology of disease, disease incidence or health of target populations at potential trial sites, and which are directly linked to clinical trials of a new diagnostic

## V Microbicides

*Research activities and processes necessary to develop and improve topical microbicides specifically intended to prevent HIV transmission; including microbicide discovery or design, preclinical and clinical development, and other activities essential for successful microbicide development and uptake*

### 20 Discovery and preclinical

*Research activities targeted at identifying, optimising, and characterising investigational microbicides and including the processes necessary to allow lead compounds to proceed to human trials including:*

20.1 Specific research aimed at discovery of topical applications for microbicide use (e.g. vaginal defence enhancers, surfactants, entry/fusion inhibitors and replication inhibitors)

20.1 Target validation, characterisation and selection

20.2 Preclinical evaluation of microbicide candidates including determination of acceptable formulation and delivery modes

20.3 Studies supporting safety & immunogenicity testing in animal models and looking at *in vitro* correlates of *in vivo* protective response

20.4 Developing reagents and standardised methods to assess microbicide-induced immune response in animals and humans

20.5 Optimisation of microbicide candidates, bioprocess development, formulation and mode of delivery of novel prevention tools for broad international use (cheap, easy to produce, stable, easy to administer) including production of GLP and GMP-grade product for regulatory toxicology studies

20.6 Preparation of an Investigational New Drug (IND) application for regulatory submission

## 21 CLINICAL DEVELOPMENT

*Activities and processes associated with clinical testing of investigational new microbicides so as to demonstrate safety and efficacy in human subjects (as needed for regulatory approval), together with other costs required to support such clinical trials, including:*

21.1 Phase I trials of novel microbicide products aimed at determining early safety and efficacy in healthy human subjects

21.2 Phase II clinical trials that evaluate safety and efficacy of the microbicide in a controlled setting, including dose-finding and proof-of-principle studies

21.3 Phase III trials of novel microbicide products aimed at confirming efficacy and safety in expanded populations

21.4 Assessment & validation of trial sites directly linked to planned product trials

21.5 Infrastructure and site development costs associated with the conduct of clinical trials for microbicide development *in endemic countries* (e.g., refurbishment of hospital wing, vehicle purchase, generators, training and community relationship building)

21.6 Further microbicide development to generate the final clinical formulation, dosage form and other Chemistry and Manufacture Control (CMC) activities required for regulatory submission

21.7 Compiling of all non-clinical and clinical data for submission of a New Drug Application (NDA) to regulatory authorities

21.8 Behavioural research *during clinical trials* relating to risk assessment, factors affecting adherence to protocol and product acceptability

21.9 Protocol development, investigator meetings, GCP-monitoring, quality control, data management, analysis and reporting, establishing a Data Safety Monitoring Board (DSMB) and trial audits

## 22 PHASE IV/PHARMACOVIGILANCE

*Studies relating to the detection, monitoring, evaluation, and prevention of adverse events associated with newly approved microbicides so as to bridge the gap between highly controlled clinical trials intended for regulatory approval and the largely uncontrolled delivery of new microbicides. Also includes studies conducted after regulatory approval that assess microbicide effectiveness in the wider population or which are necessary to support product use in developing countries.*

22.1 Pharmacovigilance and post-registration studies of newly registered microbicides to assess adverse reactions, toxicology and safety

22.2 Effectiveness studies and head-to-head comparator studies of newly registered microbicides (with other therapies or interventions)

22.3 Cost-effectiveness studies of newly registered microbicides

22.4 Treatment interactions and population level studies (of newly registered preventative microbicides e.g., pharmaco-epidemiological and resistance studies)

22.5 Behavioural research *post-registration* of new microbicides relating to risk assessment, factors affecting adherence to protocol, provider compliance, and product acceptability

22.6 Case history reports and assessment of long-term prophylaxis using newly registered microbicides in communities in endemic areas

### 23 Baseline epidemiology

*Studies evaluating potential trial site populations to confirm disease incidence, prevalence or exposure risk, and which serve as the foundation for determining the optimal collection, analysis, interpretation and presentation of clinical trial data, including:*

23.1 Epidemiological studies <u>directly</u> linked to the conduct or support of clinical trials of microbicides in development, in order to assess or validate the epidemiology of disease, disease incidence, or health of target populations at trial sites

23.2 Preliminary studies of morbidity & mortality at potential clinical trial sites, where these studies are directly linked to planned microbicide trials

## VI Therapeutic Vaccines

*Research activities and processes necessary to develop and improve investigational vaccines specifically intended to* ***treat*** *infection; including vaccine design, preclinical and clinical development, and other activities essential for successful vaccine development and uptake*

### 24 Discovery and preclinical

*Research activities targeted at discovering and optimising investigational therapeutic vaccines and including the processes necessary to allow a candidate vaccine to proceed to human trials; including:*

24.1 Studies supporting novel vaccine design including target validation & candidate optimisation

24.2 Evaluation of vaccine technologies (e.g., adjuvants, delivery systems) to improve the immunogenicity of an identified candidate

24.3 Pre-clinical safety and immunogenicity studies with candidate vaccines, including use or development of functional assays

24.4 Pre-clinical animal studies, challenge models, and studies addressing the correlation between *in vitro* models, animal models and field results

24.5 Studies on the genetics of the immune response to selected antigens as vaccine candidates, optimisation of animal models and correlates to clinical results

24.6 Manufacturing scale-up and consistency of manufacture, including production of GLP and GMP-batches for regulatory toxicology studies and other Chemistry and Manufacture Control (CMC) activities required to allow a candidate vaccine to proceed to human trials

24.7 Research on safety and regulatory considerations (e.g. validation of preclinical assays to permit registration)

24.8 Preparation of an Investigational New Drug (IND) application for regulatory submission

24.9 Optimisation of vaccine candidates for global use (cheaper, more stable, ease of administration, addition of developing country-specific targets)

## 25 CLINICAL DEVELOPMENT

*Activities and processes associated with clinical testing of investigational therapeutic vaccines so as to demonstrate safety, immunogenicity, and efficacy in human subjects (as needed for regulatory approval), together with other costs required to support such clinical trials; including:*

25.1 Phase Ia studies assessing safety, dosing and immunogenicity in human volunteers; Phase Ib studies assessing safety, dosing and immunogenicity in clinically exposed or high-risk populations

25.2 Phase IIa challenge studies; Phase II safety and preliminary efficacy studies in exposed populations or those at high-risk of infection

25.3 Phase III expanded efficacy, effectiveness and safety studies required for registration purposes, including implementation, retention and follow-up of volunteers

25.4 Assessment and validation of trial sites to carry out product trials

25.5 Infrastructure and site development costs associated with the conduct of clinical trials for vaccine development *in endemic countries* (e.g. refurbishment of hospital wing, vehicle purchase, generators)

25.6 Further biological/product development to generate the optimal clinical formulation, dosage form and other Chemistry and Manufacture Control (CMC) activities required for regulatory submission

25.7 Behavioural research *during clinical trials* relating to risk assessment, factors affecting adherence to protocol and product acceptability

25.8 Compiling of all non-clinical and clinical data to obtain a Biologics License from regulatory authorities

25.9 Protocol development, investigator meetings, GCP-monitoring, quality control, data management, analysis and reporting, establishing a Data Safety Monitoring Board (DSMB) and trial audits

## 26 PHASE IV/PHARMACOVIGILANCE

*Studies relating to the detection, monitoring, evaluation, and prevention of adverse events associated with newly approved therapeutic vaccines so as to bridge the gap between highly controlled clinical trials intended for regulatory approval and the largely uncontrolled delivery of new therapeutic vaccines. Also includes studies conducted after regulatory approval that assess therapeutic vaccine effectiveness in the wider population or which are necessary to support product use in developing countries.*

26.1 Pharmacovigilance and post-registration studies of newly registered therapeutic vaccines to assess adverse reactions, toxicology and safety

26.2 Effectiveness studies and head-to-head comparator studies of newly registered therapeutic vaccines (with other therapies or interventions)

26.3 Cost-effectiveness studies of newly registered therapeutic vaccines

26.4 Treatment interactions and population level studies (of newly registered therapeutic vaccines e.g., pharmaco-epidemiological and resistance studies)

26.5 Behavioural research *post-registration* of new therapeutic vaccines relating to risk assessment, factors affecting adherence to protocol, provider compliance, and product acceptability

26.6 Case history reports and assessment of long-term prophylaxis using newly registered therapeutic vaccines in communities in endemic areas

### 27 BASELINE EPIDEMIOLOGY

*Studies evaluating potential trial site populations to confirm disease incidence, prevalence, or exposure risk, and which serve as the foundation for determining the optimal collection, analysis, interpretation, and presentation of clinical trial data; including:*

27.1 Epidemiological studies directly linked to the conduct or support of clinical trials to assess or validate the epidemiology of disease, disease incidence or health of target populations at potential trial sites

27.2 Preliminary studies of morbidity & mortality at potential clinical trial sites necessary for product development

## VII VECTOR CONTROL PRODUCTS

### 28 PESTICIDES

*This product category ONLY includes chemical pesticides intended for global public health use and which specifically aim to inhibit and kill vectors associated with transmitting poverty-related diseases.*

*Baits, traps, predation measures, biological larvicides, habitat control and infrastructure measures are excluded from this product category.*

28.1 <u>Primary and secondary screening and optimisation</u>

*Laboratory-based design, synthesis and testing of potential insecticides, chemical larvicides, molluscides, trypanocides etc. and generation of data sufficient to allow developers to proceed field testing, including:*

28.1.1 Primary and secondary screens (e.g. *in vitro* & *in vivo* screens, chemical screens, whole insect screens)

28.1.2 Target validation and characterisation

28.1.3 Lead optimisation, synthesis optimisation

28.1.4 Early toxicology screens (e.g. acute oral toxicity, eye and skin irritation studies, AMES/mutagenicity studies)

28.1.5 Applied laboratory research and small-scale field trials, including *in vitro* & glass house efficacy testing

28.1.6 Acute toxicology and ecotoxicology studies (e.g. animal studies, exposure studies, fish and wildlife studies)

28.1.7 Metabolism and stability studies in plants and animals including mode of action, residue analysis & cross-resistance studies

28.1.8 Environmental effect and decomposition studies in soil, water & air

28.2 <u>Development</u>

*Pre-registration activities and processes associated with clinical testing of investigational chemical pesticides so as to generate data sufficient to allow developers to proceed to product roll-out & dissemination and including other costs required to support such clinical trials.*

28.2.1 Small-scale efficacy studies, residue plots and field studies necessary for product optimisation and registration

28.2.2 Acute and long-term toxicology and ecotoxicology studies

28.2.3 Metabolic and residual fate studies, crop residue and exposure data

28.2.4 Environmental assessment and environmental chemistry data

28.2.5 Generation of hazard data in humans, domestic animals and non-target plants and animals

28.2.6 Compiling of all laboratory and field data necessary for submission to regulatory authorities

28.2.7 Behavioural research conducted *pre-registration* relating to risk assessment, factors affecting adherence to protocol, and product acceptability

28.2.8 Manufacturing process development, formulation and scale-up

28.3 <u>WHOPES Evaluation</u>

*Post-registration activities that comprise entomological, safety and epidemiological evaluation (where appropriate) and development of specifications required for application of pesticide products for use in international public health programmes, including:*

28.3.1 WHOPES Phase I Laboratory studies (e.g. determining intrinsic insecticidal activity, diagnostic concentration, irritant or excito-repellent properties, cross-resistance to other insecticides, efficacy and residual activity on relevant substrates)

28.3.2 WHOPES Phase II small-scale field trials (e.g. efficacy and persistence under different ecological settings, dosage of application, handling and application, perceived side-effects)

28.3.3 WHOPES Phase III large-scale field trials (e.g. efficacy and residual activity, operational and community acceptance)

28.3.4 Assessment & validation of trial sites directly linked to product trials

28.3.5 Infrastructure and site development costs associated with the conduct of clinical trials for pesticide development *in endemic countries* (e.g. refurbishment of hospital wing, vehicle purchase, generators, training and community relationship building)

28.3.6 Behavioural research conducted *post-registration* relating to risk assessment, factors affecting adherence to protocol, provider compliance and product acceptability

28.3.7 Studies that confirm efficacy, improve product uptake or confirm safety (e.g., studies to measure impact, usage levels, contamination potential or storage and disposal needs)

28.3.8 Surveillance studies directly linked to the conduct of field trials for vector control products; including studies that determine prevalence, track distribution, abundance, or significant habits of target vectors or the vector-borne pathogen

## 29 BIOLOGICAL CONTROL PRODUCTS

*This product category ONLY includes research and development of innovative biological control interventions that specifically aim to kill or control vectors associated with transmitting poverty-related diseases (e.g. microbial/bacteriological larvicides, sterilisation techniques, and genetic modification measures)*

*Biological control interventions comprise the use of natural enemies or “engineered” products to manage vector populations either through the introduction of natural parasites, pathogens or predators of the target, or via the introduction of modified vector species to compete with natural sources*

*Baits, traps, predation measures, habitat control, chemical pesticides, and infrastructure measures are excluded from this product category.*

### 30 VACCINES TARGETING ANIMAL RESERVOIRS

*This product category ONLY includes research and development of veterinary vaccines specifically designed to prevent animal to human transmission of neglected diseases. Vaccines developed and used solely for veterinary purposes are excluded from this product category*

# VIII CANNOT BE ALLOCATED TO ONE DISEASE

### 31 CORE FUNDING OF A MULTI-DISEASE R&D ORGANISATION

*Use this category if you have disbursed core funding or non-earmarked funding to an organisation that researches and develops products for multiple neglected diseases* ***and you do not know how your funding has been allocated within that organisation***

Example:

i. Core funding has been allocated to an organisation that researches tuberculosis, buruli ulcer and leprosy but the donor does not know how much has been allocated to each disease.

### 32 ADJUVANTS AND IMMUNOMODULATORS

*Adjuvants and immunomodulators are compounds or structures that aim to improve, modulate or potentiate the immune response. These include compounds such as CpG oligonucleotides, lipopolysaccharide derivatives, Toll-like receptor agonists, chemokines and cytokines.*

*This category has* ***strict limitations****. It ONLY includes funding for R&D which meets the following conditions:*

- It is conducted by **public, philanthropic or not-for-profit entities**
- It is **basic research** i.e. it is not yet directed towards a specific disease or product. (*NB: Adjuvants and immunomodulators used in product development should be recorded elsewhere, as per the note below)*
- It is aimed at developing safer, cheaper, more immunogenic adjuvants and immunomodulators suitable for use in developing country products
- The resulting research findings or leads MUST be accessible to organisations developing pharmaceutical or biological products for neglected diseases

Examples of accepted entries in this category are:

i. Understanding the innate or adaptive immune response, e.g. studies of Toll-like receptors for the purpose of adjuvant discovery

ii. Strategies for targeting the cellular immune response to improve the quality of known adjuvants

iii. Developing a systematic approach to adjuvant discovery (e.g. predictive in vitro assays)

iv. High throughput screening to identify potential adjuvants

How to record funding for adjuvants and immunomodulators being developed as part of a product

If the adjuvant or immunomodulator is being developed as part of a product, then the investment should be recorded under the relevant disease and product. For example, if the adjuvant R&D is as part of a malaria vaccine construct, then enter it under Malaria (in Step 1) and Vaccines (in Step 2).

## 33 DELIVERY TECHNOLOGIES AND DEVICES

*Delivery technologies and devices comprise mucosal delivery tools, vaccine carrier systems, and alternative delivery technologies that facilitate the successful delivery of pharmaceutical or biological products in a resource-limited setting (e.g. dendritic cell systems, emulsions, novel viral vectors, sprays, patches and needle-free devices).*

Examples:

i. Development of an intra-nasal vaccine delivery platform or the development of a plant-based sub-unit vaccine delivery system
ii. Discovery and optimisation of mechanisms for controlled release

## 34 GENERAL DIAGNOSTIC PLATFORMS

*This category has* ***strict limitations.*** *It ONLY includes funding for R&D which meets the following conditions:*

- It is conducted by **public, philanthropic or not-for-profit entities**
- It is basic research i.e. **it is not yet directed towards a specific disease or product**
    - o If a project has progressed to the product development stage, please go back to Steps 1 and 2 and select the specific disease and product category where the diagnostic platform will be used (e.g. TB diagnostics, HIV diagnostics) before entering your data
- Research is aimed at developing cheaper, faster, more user friendly diagnostic platforms or technologies**,** intended for use in resource-limited settings

Examples

i. Simplification of a diagnostic approach to reduce costs e.g. lateral flow tests for malaria
ii. Re-designing tests/ equipment to use cheap, standard reagents
iii. Work to make standard tests cheaper and simpler e.g. simpler ELISA's; simpler Nucleic Acid Amplification tests; simpler DST's

## 35 OTHER R&D

*Use this category for funding disbursed or received for research and development efforts that simultaneously focus on two or more neglected diseases, so that* ***funds cannot be apportioned to the specific disease categories.***

Examples:

i. Research into the interaction between HIV and tuberculosis
    - If the research is part of the normal development path of a product for a specific disease (e.g testing new TB drugs in special populations such as AIDS patients), please go back to Steps 1 and 2 and select the specific disease and product category where the product will be used (e.g. TB drugs)
ii. Development of a diagnostic to differentiate between different causes of fever (e.g., between malaria and meningitis)
iii. Development of a multi-disease diagnostic (e.g. the test can identify tuberculosis, malaria or sleeping sickness depending on the reagents used)
iv. Development of a multi-disease vaccine e.g. a combination pneumonia/ meningitis vaccine

*IF funding CAN be assigned to the specific disease categories, please enter this information under the appropriate disease and product categories provided.*

# IX OUT OF SCOPE (EXCLUDED FROM THE SURVEY)

## 36 GENERAL EXCLUSIONS

The G-FINDER survey is only capturing investments that support pharmaceutical R&D aimed at preventing, treating or curing neglected diseases for patients in developing countries. The following are excluded from the scope:

36.1 Non-pharmaceutical tools

36.1.1 Adult male circumcision, cervical barriers, HSV-2 prevention, bednets, traps, water sanitation tools

36.2 General supportive, nutritional and symptomatic therapies

36.2.1 Oral rehydration therapy

36.2.2 Micronutrient supplementation, vitamins

36.2.3 Anti-pyretics, painkillers

36.3 Products developed and used for veterinary purposes are excluded

36.4 In-kind contributions

36.4.1 In-kind R&D contributions are excluded from the survey due to the difficulty in quantifying their value; however a sample of these contributions will be highlighted in the final report. Typical in-kind contributions would include e.g. training of developing country scientists, sharing of expertise, access to compounds

36.5 Additional exclusions for private sector investment

36.5.1 Industry overhead costs, capital costs and opportunity costs are excluded, due to the difficulty of quantifying these and allocating them to the neglected disease investment. However, the published report will acknowledge the role of these in contributing to costs.

## 37 NON-PRODUCT R&D

*Our intention is to capture investments into **neglected disease product development** as accurately as possible. Therefore, the following R&D activities are excluded from the survey.*

37.1 Clinical studies that are not linked to development of a NEW product

37.1.1 Protocol studies and clinical trials using established, available products (not linked to formal label-extension trials of new products).

37.1.2 Epidemiological surveillance and monitoring studies that are not directly linked to product development. For example, routine DSS (Demographic Surveillance System) activities.

37.2 Health services and access research

37.2.1 Any clinical study not linked to development of a product products – disease management, studies of community attitudes, knowledge and practice in relation to neglected disease treatment and control programs

37.2.2 Health care service studies in relation to delivery of neglected disease treatment and control measures

37.2.3 Design of treatment and control programs appropriate to local prevailing conditions

37.2.4 Implementation and evaluation of large-scale neglected disease treatment and control programs operated through health care services, government ministries, nongovernmental organizations etc

37.2.5 Roll-out of proven vector control products (e.g. traps and nets, DDT)

37.2.6 Advocacy, community education and policy activities related to use, access, or roll-out of new products

37.3 Operational Programme Assessment

37.3.1 Reviews on the status of neglected disease product development

37.3.2 Studies on the economic impact of neglected disease morbidity and mortality on communities

37.3.3 Studies on the economics of neglected disease prevention and control measures

37.3.4 Mathematical modelling of the disease (e.g. transmission, immune response)

37.3.5 Fostering collaboration between academia, industry, government agencies, and NGOs

37.4 GENERAL Capacity Building (human & infrastructure)

*Capacity building activities are excluded except those that are DIRECTLY linked to development of a new neglected disease product. The following activities are therefore excluded:*

37.4.1 Building academic research capacity; improving existing academic capacity (except as directly linked to development of a specific product)

37.4.2 Providing training opportunities; strengthening R&D institutional capacity; developing and maintaining personnel (except as directly linked to development of a specific product)

37.4.3 Major infrastructure development (e.g. design, construction and validation of large-scale manufacturing facilities)

## 38 SELECTED DISEASE AND PRODUCT RESTRICTIONS

*Commercial diseases where incentives for R&D already exist; or product R&D already occurs in response to the existing Western markets are EXCLUDED from this survey.*

38.1 Basic research

*R&D for Basic Research is LIMITED for the following diseases:*

38.1.1 HIV/AIDS

ONLY includes Basic mechanisms related to preventative vaccines and microbicides (e.g. immunology responses to potential antigens, mechanism of mucosal transmission)

38.1.2 Multiple Diarrhoeal diseases

*Basic research is EXCLUDED for diseases in which there is a commercial market for ANY product as we assume investments into basic research will be driven by that market.*

*Basic research is therefore excluded for the following diseases:*

38.1.3 Bacterial pneumonia & meningitis

38.1.4 Trachoma

38.1.5 Rheumatic fever

38.2 Drugs

*R&D for drugs is LIMITED for the following diseases:*

38.2.1 HIV/AIDS

ONLY includes label extensions[2] and reformulations for developing country use (e.g. paediatric or slow-release formulations).

38.2.2 Diarrhoea caused by Cholera, Shigella, cryptosporidium

ONLY includes pharmacological interventions that target the pathogen. Supportive therapies (e.g. zinc treatment, oral rehydration therapy, or other fluid and nutritional supplements) are EXCLUDED.

*R&D for drugs is EXCLUDED for the following diseases:*

38.2.3 Bacterial pneumonia & meningitis

38.2.4 Diarrhoea (except Cholera, Shigella, cryptosporidium)

38.2.5 Trachoma

38.2.6 Rheumatic fever

38.2.7 Typhoid and paratyphoid fever

38.3 <u>Preventive Vaccines</u>

*R&D for preventive vaccines is LIMITED for the following diseases:*

38.3.1 Bacterial pneumonia caused by *S. pneumoniae*

ONLY includes R&D on vaccines specifically for developing-country registration. Such a vaccine must at a minimum: a) be designed for use in infants less than two years of age; and b) provide coverage against *S. pneumoniae s*erotypes 1, 5, and 14.

For multi-valent vaccines covering Western and developing country strains, only developing country-specific costs should be entered; including for trials, registration and Phase IV/pharmacovigilance studies.

38.3.2 Bacterial pneumonia or meningitis caused by *N. meningitidis*

ONLY includes R&D on vaccines specifically for developing-country registration. Such a vaccine must, at a minimum: a) provide coverage against *N. meningitidis* serotype A; b) be a conjugate vaccine; c) be designed for use in infants less than two years of age; and d) be designed to cost less than a dollar per dose.

For multi-valent vaccines covering Western and developing country strains, only developing country-specific costs should be entered; for example, for trials, registration and Phase IV/pharmacovigilance studies in the target developing countries.

38.3.3 Diarrhoea caused by rotavirus

ONLY includes developing country-specific R&D, including clinical trials, registration and Phase IV/pharmacovigilance studies in the target developing countries.

*R&D for preventive vaccines is EXCLUDED for the following diseases:*

38.3.4 Diarrhoea caused by Giardia

38.3.5 Roundworm

38.3.6 Whipworm

38.3.7 Tapeworm (Cysticercosis/Taeniasis)

38.3.8 Lymphatic Filariasis (elephantiasis)

38.3.9 Leprosy

[2] <u>Label-extensions</u> refer to changes to drugs or their labels after they have been approved. This includes changes in manufacturing, recommended patient population, and/or formulation. To change a label, market a new dosage or strength of a drug, or change the way a drug is manufactured, the Company must submit a supplemental new drug application (sNDA) to regulatory authorities to obtain marketing approval.

### 38.4 Diagnostics

*R&D for diagnostics is LIMITED to the development of cheap, stable, easy-to-use diagnostics suited to resource-limited settings.*

*R&D for diagnostics is EXCLUDED for the following diseases:*

- 38.4.1 Diarrhoea caused by rotavirus
- 38.4.2 Roundworm
- 38.4.3 Hookworm
- 38.4.4 Whipworm
- 38.4.5 Tapeworm (Cysticercosis/Taeniasis)
- 38.4.6 Lymphatic Filariasis (elephantiasis)
- 38.4.7 Rheumatic fever

### 38.5 Vaccines (Therapeutic)

*R&D for therapeutic vaccines is EXCLUDED for the following diseases:*

- 38.5.1 HIV/AIDS
- 38.5.2 Malaria
- 38.5.3 Bacterial pneumonia and meningitis
- 38.5.4 Diarrhoea
- 38.5.5 Sleeping sickness
- 38.5.6 Buruli ulcer
- 38.5.7 Dengue
- 38.5.8 Helminths (except Schistosomiasis)
- 38.5.9 Leprosy
- 38.5.10 Rheumatic fever
- 38.5.11 Trachoma
- 38.5.12 Typhoid and paratyphoid fever

### 38.6 Microbicides

Applications that may have Western markets or be useful for other STDs (e.g. mucosal delivery technology, adjuvants) are EXCLUDED.

### 38.7 Vector Control Products

#### 38.7.1 Pesticides

This product category ONLY includes chemical pesticides intended for global public health use and which specifically aim to inhibit and kill vectors associated with transmitting poverty-related diseases.

Baits, traps, predation measures, biological larvicides, habitat control and infrastructure measures are excluded from this product category.

#### 38.7.2 Biological Control Products

This product category ONLY includes research and development of innovative biological control interventions that specifically aim to kill or control vectors associated with transmitting poverty-related diseases (e.g. microbial/bacteriological larvicides, sterilisation techniques, and genetic modification measures)

Baits, traps, predation measures, habitat control, chemical pesticides, and infrastructure measures are excluded from this product category.

38.7.3 Vaccines targeting animal reservoirs

This product category ONLY includes research and development of veterinary vaccines specifically designed to prevent animal to human transmission of neglected diseases.

Vaccines developed and used solely for veterinary purposes are excluded from this product category

## 38.8 Cannot be allocated to one disease

38.8.1 Adjuvants and immunomodulators

This category has **strict limitations**. It ONLY includes funding for R&D which meets the following conditions:

- It is conducted by **public, philanthropic or not-for-profit entities** (i.e. private sector investment into adjuvants and immunomodulators is EXCLUDED)
- It is upstream research that is **not yet directed towards a specific disease or product**.
    - If a project has progressed to the product development stage, please go back to Steps 1 and 2 and select the specific disease and product category where the adjuvant or immunomodulator will be used (e.g. TB vaccine, HIV vaccine) before entering your data.
- Research is aimed at developing safer, cheaper, more immunogenic adjuvants and immunomodulators.
- The resulting research findings or **leads must be accessible** to organisations developing pharmaceutical or biological products intended to treat or prevent neglected diseases.
- If a project has progressed to the product development stage, please go back to Steps 1 and 2 and select the specific disease and product category where the adjuvant or immunomodulator will be used (e.g. TB vaccine, HIV vaccine) before entering your data.

38.8.2 General diagnostic platforms

This category has **strict limitations.** It ONLY includes funding for R&D which meets the following conditions:

- It is conducted by **public, philanthropic or not-for-profit entities** (i.e. private sector investment into adjuvants and immunomodulators is EXCLUDED).
- It is upstream research which is **not yet directed towards a specific disease or product.**
    - If a project has progressed to the product development stage, please go back to Steps 1 and 2 and select the specific disease and product category where the diagnostic platform will be used (e.g. TB diagnostics, HIV diagnostics) before entering your data.
- Research is aimed at developing cheaper, faster, more user friendly diagnostic platforms or technologies**,** intended for use in resource-limited settings.
