# Supplementary material for: Neglected Disease Research and Development: How Much Are We Really Spending?
Source: PLoS Med. 2009 Feb 3;6(2):e1000030. doi: 10.1371/journal.pmed.1000030 (PMC2634791; doi:10.1371/journal.pmed.1000030)
Supplement: Supplementary file 2 [file pmed.1000030.sd001.doc]

| Member | Organisation | Title |
| --- | --- | --- |
| Regina Rabinovich | Bill & Melinda Gates Foundation | Director of Infectious Diseases |
| Carole Heilman | National Institute of Allergy and Infectious Diseases (NIAID) | Director of Division of Microbiology and Infectious Diseases (DMID) |
| Ted Bianco | Wellcome Trust | Director of Technology Transfer |
| Giorgio Roscigno | Foundation for Innovative New Diagnostics (FIND) | Chief Executive Officer |
| Robert Ridley | WHO - Special Programme for Research and Training in Tropical Diseases (TDR) | Director |
| Marie-Paule Kieny | WHO - Initiative for Vaccine Research (IVR) | Director |
| Wayne Koff | International AIDS Vaccine Initiative (IAVI) | Senior Vice President of Research and Development |
| Timothy Wells | Medicines for Malaria Venture (MMV) | Chief Scientific Officer |
| Lewellys F. Barker | Aeras Global TB Vaccine Foundation | Senior Medical Advisor |
| Mel Spigelman | The Global Alliance for TB Drug Development (TB Alliance) | Director of Research and Development |
| Simon Croft | London School of Hygiene & Tropical Medicine | Professor of Parasitology |
| Michael J Free | Program for Appropriate Technology in Health (PATH) | Vice President and Senior Advisor for Technologies |
| Peter Hotez | George Washington University (GWU) | Walter G. Ross Professor and Chair of the Department of Microbiology, Immunology and Tropical Medicine |
| N K Ganguly | Department of Health Research, Union Ministry of Health and Family Welfare | Secretary |
| Ripley Ballou | GlaxoSmithKline Biologicals | Vice President, Emerging Diseases,  Clinical Research and Development |
| Joseph Romano | International Partnership for Microbicides (IPM) | Executive Director for Research and Development |
| *Janet Hemingway* | Innovative Vector Control Consortium (IVCC) | Chief Executive Officer |
