# Supplementary material for: Neglected Disease Research and Development: How Much Are We Really Spending?
Source: PLoS Med. 2009 Feb 3;6(2):e1000030. doi: 10.1371/journal.pmed.1000030 (PMC2634791; doi:10.1371/journal.pmed.1000030)
Supplement: Supplementary file 3 [file pmed.1000030.sd002.doc]

Organization

AstraZeneca

Bill and Melinda Gates Foundation

Brazil Ministry of Health, Department of Science and Technology

Crucell

Eli Lilly and Company

European Commission: Research Directorate-General

GlaxoSmithKline (GSK)

Irish Aid

Merck and Co Inc

Netherlands Ministry of Foreign Affairs - Directorate General of Development Cooperation (DGIS)

Novartis International AG

Otsuka

Pfizer

Public Health Agency of Canada (PHAC)

Sanofi Aventis

South Africa Department of Science and Technology (DST)

Swiss Agency for Development and Cooperation (SDC)

UK Department for International Development (DfID)

UK Medical Research Council (MRC)

United States Agency for International Development (USAID)

US Centers for Disease Control (CDC)

US Department of Defense

US National Institutes of Health (NIH)

Wellcome Trust

*Wyeth-Ayerst Lederle, Inc*
