# Supplementary material for: Neglected Disease Research and Development: How Much Are We Really Spending?
Source: PLoS Med. 2009 Feb 3;6(2):e1000030. doi: 10.1371/journal.pmed.1000030 (PMC2634791; doi:10.1371/journal.pmed.1000030)
Supplement: Supplementary file 4 [file pmed.1000030.sd004.doc]

**3 Step Process: 1 - Select Disease**

**3 Step Process: 2 - Select Product**

**3 Step Process: 3 - Select R&D Area**

**Table: Entering data**

**Grants that cannot be allocated to one disease: First step - drop-down selections**

**Grants that cannot be allocated to one disease: Second step - R&D Reference Document pop up to guide correct allocation**
